# The genetic and epigenetic association of LDL Receptor Related Protein 1B (*LRP1B*) gene with childhood obesity

**DOI:** 10.1038/s41598-019-38538-2

**Published:** 2019-02-12

**Authors:** Suman Lee

**Affiliations:** Division of Genomic Research, Center for Genome Science, National Institute of Health, Chungcheongbuk-do, 363-951 Republic of Korea

## Abstract

Low-density lipoprotein Receptor Related Protein 1B (*LRP1B*) is homologous to the gigantic lipoprotein receptor-related protein 1 that belongs to the family of Low-density lipoprotein receptors. Previous genetic association studies of the *LRP1B* gene have shown its genetic association with obesity. Through exome sequencing of the *LRP1B* gene from a childhood severe obesity cohort (n = 692), we found novel single nucleotide polymorphism (rs431809) in intron 4, which has been significantly correlated with both body mass index (BMI) and waist-hip-ratio (WHR). Three methylations of CpG sites (cg141441481, cg01852095 and cg141441470) in the same intron were also significantly correlated with BMI and WHR. All CpG methylations had bimodal patterns, and were dependent on rs431809 genotypes. The genetic influences of obesity on the *LRP1B* gene may be linked to the interplay of CpG methylations in the same intron. Heritability of SNP interacts with epigenetic crosstalk in *LRP1B*. Genetic and epigenetic crosstalk of *LRP1B* gene may be implicated in the prevention and therapeutic approach to childhood obesity.

## Introduction

Low-density lipoprotein Receptor Related Protein 1B (*LRP1B*) is highly homologous to the gigantic lipoprotein receptor-related protein 1 that belongs to the family of low-density lipoprotein receptors (LRP)^1^. LRP is widely expressed in several tissues and plays important roles in lipoprotein catabolism, blood coagulation, cell adhesion and migration, neuronal process outgrowth, and the pathogenesis of Alzheimer’s disease^2–4^. Most of the newly identified LRP1B ligands are well-known factors in blood coagulation and lipoprotein metabolism, suggesting a possible role of *LRP1B* in atherosclerosis^5^.

Genetic association studies of novel obesity-related gene variants in large populations have reported *LRP1B* as a factor in obesity susceptibility^6–9^. For example, intergenic rs2890652 single nucleotide polymorphism (SNP) of *LRP1B* has been associated with a BMI increase of 0.09 kg/m^2^ with a *P* value of 1e-10^6^. In a genome-wide association study (GWAS) of body mass index (BMI) in Japanese people (n = 173,430), intronic SNP of *LRP1B* was shown to be significantly associated with BMI^8^. Another GWAS study in European ancestry cohorts showed that rs7583748 of *LRP1B* was strongly associated with BMI^10^. Burgdorf *et al*. reported that the rs2890652 C allele of *LRP1B* was associated with insulin resistance in a population-based sample of 6,039 Danish individuals^11^. A study examining the effect of genetic obesity loci on cognitive restraint and uncontrolled and emotional eating showed that the BMI-increasing variant of fat mass and obesity-associated protein (*FTO*) was positively associated with cognitive restraint while *LRP1B* was inversely associated with cognitive restraint^12^.

*LRP1B* is also candidate involved in the pathogenesis of cancer^13–18^. In one study, *LRP1B* mutation was a predictive marker for the presence of chronic obstruction pulmonary disease in patients with lung adenocarcinoma^18^. *LRP1B* is a novel candidate tumor suppressor gene that is inactivated by genetic and transcript alterations in nearly 50% of non-small-cell lung cancer cell lines.

Epigenetic regulation of *LRP1B* results in changes to the tumor environment, such as the aberrant methylation of *LRP1B* in acute lymphoblastic leukemia and in gastric cancer^13,14,16^. In colon cancer tissues, downregulation of *LRP1B* inhibits the growth, migration and metastasis of colon cancer cells^17^.

Here, we investigated the genetic polymorphisms of *LRP1B* in obesity by exome sequencing in a childhood obesity sample. We found an intronic SNP which was associated with Body Mass Index (BMI) and waist-hip-ratio (WHR). To understand the functional role of SNP located in this intron, we also investigated nearby CpG methylations (cg141441470, cg01852095 and cg141441481) for obesity.

In this study, we investigated the integrated effect of genetic and epigenetic changes in childhood obesity. We performed the genetic and epigenetic association study of *LRP1B* gene with obesity-related traits, as well as the sequence dependent methylation patterns linked to childhood obesity.

### Experimental Procedures

All methods are approved by the committee of Korea National Institute of Health (KNIH) and carried out in accordance with relevant guidelines and regulations of KNIH. All the analysis was performed anonymously.

### Study subjects

Study subjects were selected from the Korean Child-Adolescent Cohort Study, which followed a cohort of Korean students annually, at four elementary schools in Gwacheon, from entry into school at age 7, in 2005^19^. The overall objective of the cohort study was to identify early risk factors for obesity and associated metabolic diseases in Korean children, and details have been published previously^20^. Severe childhood obesity is defined as a BMI-for-age of ≥1.2 times the 95^th^ percentile value or above a BMI of ≥35 kg/m^2^ according to the recently proposed definition by the Centers for Disease Control and Prevention. For analysis, we used DNA samples from 305 children (159 males) with severe obesity (average age: 13.94 ± 0.84, average BMI: 31.71 ± 3.94 kg/m^2^) and from 387 lean controls (199 males) (average age: 13.93 ± 0.77, average BMI: 19.44 ± 1.34 kg/m^2^). Informed consents were obtained from parents of participants, and IRB of Korea National Institute of Health was approved (IRB approved number, 2014-08EXP-05-P-A). Detailed subject criteria are described in Table 1.

**Table 1:** Summary of the population characteristics for childhood obesity.

Variables	Controls	Cases	*P*
Age	13.93 ± 0.77	13.94 ± 0.84	0.6805
N (M/F)	387 (199/188)	305 (159/146)	0.8784
BMI	19.44 ± 1.34	31.71 ± 3.94	**<0**.**0001**
WHR	0.76 ± 0.05	0.89 ± 0.06	**<0**.**0001**

N(M/F): Number (Male/Female), BMI (kg/m2): Body Mass Index, WHR: Waist to Hip Ratio, *P* values < 0.05 are described as bold characters.

### Exome Sequencing

All exons were captured by the SureSelect Human Exon V4 (Agilent Technologies, Carlsbad, CA, USA) according to the manufacturer protocols. The captured DNA was sequenced on the Illumina HiSeq. 2500. The sequence data were assembled using the UCSE Genome Browser (GRCh37/hg19) and mapped by BWA (http://bio-bwa.sourceforge.net). Variant detection was performed using SAMTOOLS (http://samtools.sourceforge.net). An average depth of coverage was ~67.2 fold^21^.

### Quantitation of CpG DNA methylation and genotyping by pyrosequencing analyses

**Figure 1:**
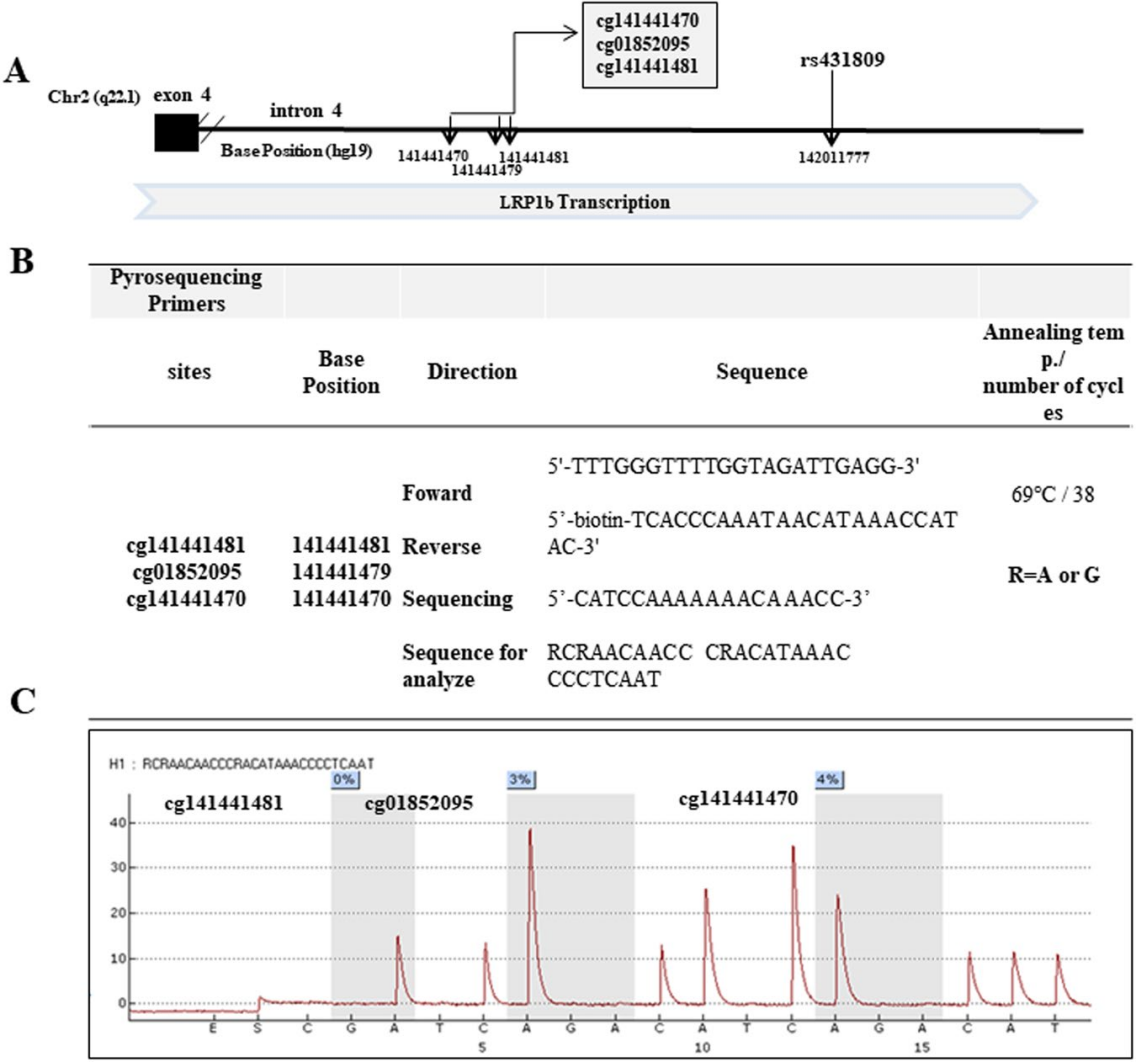
The schematic diagrams of *LRP1B* locus and Pyrosequencing. (**A**) The schematic diagrams of *LRP1B* locus with rs431809 and three CpG sites (cg141441470 cg01852095 and cg141441481) in intron 4 is diagramed. The biological position of each site was indicated by arrows. (**B**) The primer sequences and PCR conditions for pyrosequencing. (**C**) The pyrogram for the experiment was shown.

Polymerase chain reactions (PCRs) of *LRP1B* were performed for the purpose of pyrosequencing. The genomic position and gene positions of three target CpG sites analyzed via pyrosequencing are described in Fig. 1A. The PCR cycling conditions and the sequences for all primers designed by MethPrimer (Biotage AB) for cg141441470, cg01852095 and cg141441481 are described in Fig. 1B. Pyrosequencing assays were designed, optimized, performed on the PSQ HS 96 A System (Biotage AB) according to the manufacturer’s specifications (Pyrosequencing, Westborough, MA, USA). We measured the quantitation of cg141441470, cg01852095 and cg141441481 methylations. Pyrosequencing was conducted using bisulfite treated DNA. Three CpG methylation sites are located 570 kb upstream from rs431809. Technical controls for pyrosequencing revealed median methylations of 0~10% (unmethylated control), and >90% (methylated control), thereby defining detection limits of EpiTect PCR control DNA set (QIAGEN, USA). Finally, we measured DNA methylations 638 samples (numbers of controls:386, numbers of cases:252). Fifty-four samples were failed for pyrosequencing.

### Statistical analyses for genetic association study

For genetic association study, quality control was implemented in PLINK^22^. Single nucleotide polymorphisms (SNPs) were excluded for any of the following reasons: (1) minor allele frequency (MAF) < 1%; (2) genotype call rate <99%; and (3) Hardy–Weinberg equilibrium (HWE) *P* value < 1e−03. Rare genetic variants (MAF < 1%), were excluded to test for association study, because of underpowered by small sample size. Before frequency and genotyping pruning, there are 235 SNPs. 3 markers to be excluded based on HWE test (*P* <  = 1e-3). Total genotyping rate in remaining individuals is 0.999256. 4 SNPs failed missingness test (GENO > 0.01). 170 SNPs failed frequency test (MAF < 1%). After frequency and genotyping pruning, there are 61 SNPs. A genetic association was tested using the additive model in PLINK^22^. Age and sex were used as the covariates for the study performed with linear and logistic regression analysis. Raw *P* values < 0.05 was considered statistically significant.

### Statistical analyses for DNA methylation study

Statistical analysis for DNA methylation study was performed by non-parametric methods because of bimodal data distribution. Wilcoxon signed-rank test, Kruskal–Wallis test and Spearman’s correlation test were adopted. Age and sex were used as the covariates for the study performed with regression analysis. Statistical analysis was performed using R version 3.2.1 and data was visualized using ggplot2.

## Results

### Genetic association study of rs431809 with obesity related traits

The general characteristics of obese children and controls are summarized in Table 1. Childhood obese cases (n = 305) with an average BMI of 31.71 ± 3.94 and lean controls (n = 387) with an average BMI of 19.4 ± 1.34 were categorized. There was no significant difference between the two groups in age (*P* > 0.05). The average of waist-hip-ratio (WHR) were also listed with significant differences between cases and controls in Table 1.

We performed exome sequencing of *LRPB1* gene from 692 blood samples to find any genotype which associated with BMI and WHR. Before frequency and genotyping pruning, exome sequencing showed 235 single nucleotide variants existed at *LRP1B* (chr2: 140,988,992 - 142,889,270, hg19). After pruning, 61 SNPs were left for the analysis. The pruning criteria were described in details at Materials and Methods. By linear regression analysis of 61 SNPs with BMI and WHR, two SNPs for each trait were significant (raw *P* values < 0.05). Only rs431809 was significantly associated with both traits (Table 2). By case-control analysis with 387 controls and 305 obese cases, rs431809 was significantly associated with childhood obesity, with an odd ratio of 1.3165 (raw *P* values = 0.03). Age and sex were used as covariates for the study.

**Table 2:** The genetic association of *LRP1B* gene with childhood obesity.

Traits	CHR	SNP	BP	A1	Annotation	MAF	NMISS	*Beta*	STAT	P
BMI	2	rs431809	142012027	T	intron	0.1792	692	0.9514	2.034	**0**.**0423**
	2	2-141294285	141294285	A	intron	0.0419	692	−2.056	−2.233	**0**.**0258**
WHR	2	rs431809	142012027	T	intron	0.1792	692	0.01174	2.035	**0**.**0422**
	2	2-141215252	141215252	A	intron	0.0108	692	0.04636	2.148	**0**.**0321**

CHR: chromosome number, BP: Physical position (base-pair), A1: Minor allele name, MAF: Minor allele frequency, NMISS: Number of non-missing individuals included in analysis, *Beta*: regression coefficient, STAT: Coefficient t-statistic, raw *P* values < 0.05 are described as bold characters.

**Figure 2:**
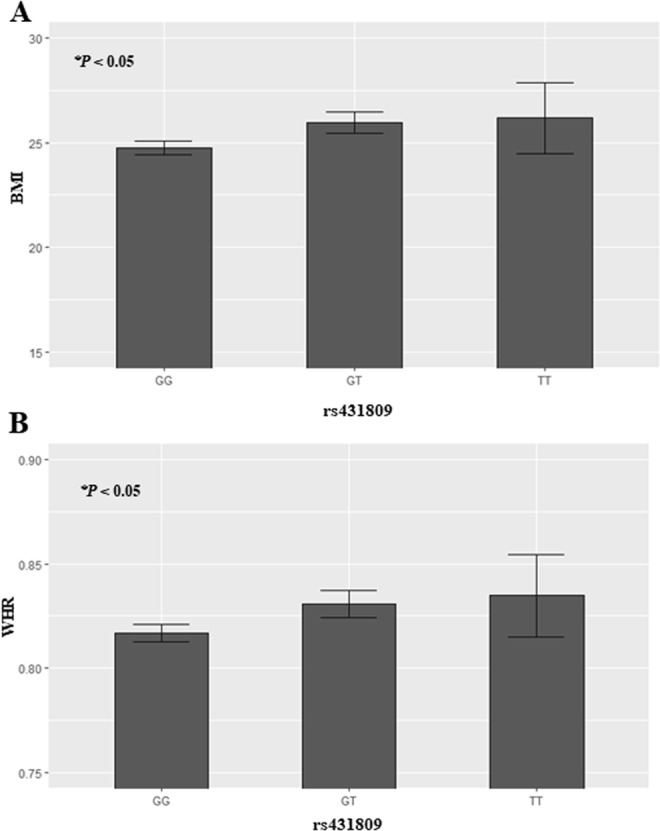
The bar graph of BMI and WHR depending on rs431809 genotype, GG (homozygous for major genotype), GT (heterozygous genotype), TT (homozygous for minor genotype). (**A**) BMI, (**B**) WHR. The statistical significance by ANNOVA test was indicated on the left top. The statistic significances below *P* value < 0.05 were also marked as a star (*).

The rs431809 was located in intron 4 and had minor allele frequency was 0.1792. Major dominant genotype (GG) was 67.4%, heterozygous genotype (GT) was 29.2% and minor recessive genotype (TT) was 3.3% in all subjects (n = 692). According to rs431809 genotypes, BMI and WHR were graphed at Fig. 2. An analysis of variance test (ANOVA) of the data presented in each bar graph showed that BMI (Fig. 2A) and WHR (Fig. 2B) significantly differed in the three groups according to genotype (*P* < 0.05).

The relationship of BMI and WHR with the rs431809 genotypes (GG: homozygous for the major genotype, GT: heterozygous, TT: homozygous for the minor genotype) are graphed in Fig. 2. There were significant changes of BMI and WHR by rs431809 with positive directions to minor genotype (TT), respectively. The differences of BMI and WHR between GG genotype to TT genotype were 1.4 kg/m^2^ and 0.018 (Fig. 2). rs431809 TT is minor genotype, and so minor genotype has tended to have higher BMI and WHR.

### CpG methylation of childhood obesity

**Figure 3:**
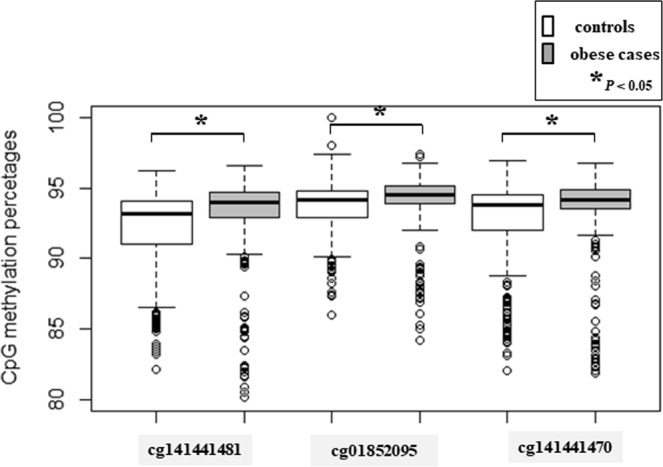
Boxplot of DNA methylation of Korean childhood obesity (n = 637). CpG methylations between the control group and the case group are compared at 3 CpG sites (cg141441481, cg01852095 and cg141441470). Gray boxes indicate obese cases. *P* values < 0.05 are also marked with a star (*).

Mechanistic studies for rs431809 genetic association with obesity are being carried out by the investigation of CpG methylations in same intron which may be important for gene regulation. We examined three CpG sites (cg141441481, cg01852095 and cg141441470) in an intron 4 of *LRP1B* by pyrosequencing using bisulfite treated DNA. The methylations between the controls and the obese cases of each CpG site were graphed at cg141441481, cg01852095 and cg141441470 sites in Fig. 3. Hypermethylation from the obese group was significant in cg141441481, cg01852095 and cg141441470 (Wilcoxon test, *P* < 0.05). Our case-control study found that three CpG methylations were hypermethylated in obese cases by 0.76% in cg141441481, 0.34% in cg01852095, and 0.97% in cg141441470 (Fig. 3).

We interrogated the CpG methylations with obesity related traits (n = 638). The regression values with beta and *P* values of the methylation of cg141441481, cg01852095 and cg141441470 with BMI and WHR were listed in Table 3, respectively. All three CpG methylations were significantly association with BMI and WHR (*P* < 0.05). The correlation of CpG methylations with BMI and WHR was all positive, which indicate hypermethylation drive the increase of obesity related traits (Table 3).

**Table 3:** Linear regression analysis of three CpG methylations of *LRP1B* gene with BMI and WHR.

		cg141441481	cg01852095	cg141441470
BMI	***Beta***	0.3531	0.2783	0.2716
	***P***	***0***.***0003***	***0***.***0475***	***0***.***009***
WHR	***Beta***	0.004	0.0036	0.0031
	***P***	***5***.***09e-11***	***4***.***23e-09***	***1***.***56e-09***

BMI: Body Mass Index, WHR: Waist to Hip Ratio, *Beta*: regression coefficient, Age and sex were used as covariates. *P* values < 0.05 are described as bold characters.

### The relationship of the CpG methylation with the rs431809 genotypes in intron 4 of *LRP1B* gene

**Figure 4:**
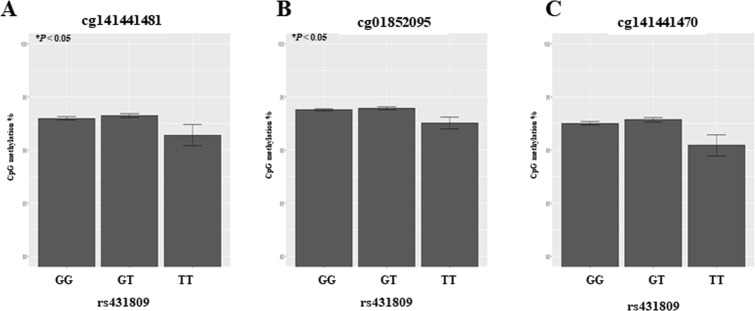
The distribution of 3 CpG methylations by the genotype of rs431809. A diagram of the distribution of DNA methylation percentages of 3 CpG sites depending on the genotypes of rs431809, GG (homozygous for major genotype), GT (heterozygous genotype), TT (homozygous for minor genotype). The methylation percentage of (**A**) cg141441481 (**B**) cg01852095 (**C**) cg141441470 depending on the rs431809 genotypes.

To investigate genetic and epigenetic interaction, we further analyzed three CpG methylations (cg141441481, cg01852095 and cg141441470) in intron 4 of *LRP1B* gene. The relationship of the CpG methylation with the rs431809 genotypes (GG: homozygous for the major genotype, GT: heterozygous, TT: homozygous for the minor genotype) are graphed in Fig. 4. CpG methylations were altered dependent on the genotype of rs431809. cg141441481 and cg01852095 methylations significantly depended on rs431809 genotypes (Kruskal–Wallis test, *P* < 0.05), but not cg141441470. The methylation changes of three CpG sites from GG genotype to TT genotypes were the negative direction (hypomethylation) in all CpG sites, respectively (Fig. 4A–C). The difference of CpG methylation between GG genotype to TT genotype were 2.07% in cg141441481, 1.23% in cg01852095 and 1.58% in cg141441470 on the average.

### The correlation significance and strength of the CpG methylation with BMI differ in rs431809 genotype

**Figure 5:**
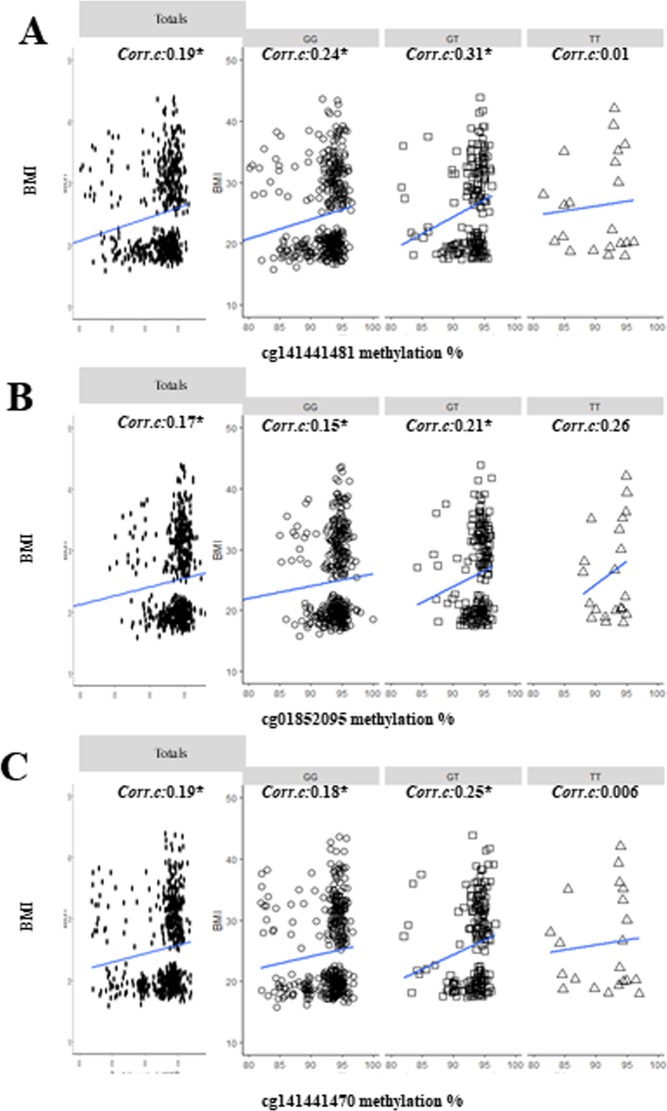
Correlations of three CpG methylations with BMI, by rs431809 genotype, TT: homozygous major genotype, GT: heterozygous genotype, GG: homozygous minor genotypes. In (**A**) cg141441481 (**B**) cg01852095 (**C**) cg141441470 methylation in totals, GG, GT, TT group. Correlation lines fitted on graphs with correlation coefficients (*Corr*.*c*) indicated in the top of each graph. The statistic significances below *P* value < 0.05 were also marked as a star (*).

We investigated if the correlation of CpG methylations with BMI, would be dependent on rs431809 genotypes. BMI significantly correlated with cg141441481 (Fig. 5A), cg01852095 (Fig. 5B), and cg141441470 (Fig. 5C) in all subjects (Spearman test, *P* < 0.05). Correlational strengths were similar and positive, in all CpG sites, 0.19 in cg141441481, 0.17 in cg01852095 and 0.19 in cg141441470.

We investigate CpG methylation was different on the subject’s genotype in the same intron. After dividing by rs431809 genotype, strength and significance of correlations differed by genotype (Fig. 5A–C). For correlation coefficients of GG (n = 430), GT (n = 187) and TT (n = 21) group, three CpG methylations significantly correlated with BMI in both GG and GT group. In TT group, the correlations with BMI were insignificant in three CpG sites (Spearman test, *P* > 0.05), and very low in cg141441481 and cg141441470 (*Corr*.*c* ≤ 0.01). Small sample size (n = 21) and lower mean value of CpG methylation in TT group may affect the significance and strength of correlation. Each CpG methylation may have different genetic dependency of the rs431809 genotype, respectively.

## Discu**ss**ion

Previous genetic association study of *LRP1B* showed strong association with obesity^6–8,10,11^. In this study, we identified novel BMI-associated SNP, rs431809. We compared the effect size of rs431808 with those of previously reported BMI-related SNPs of *LRP1B* gene. Effect size of rs431809 in our severe childhood obesity study was larger as 0.95 kg/m^2^ BMI increase compared with intronic rs12617004 with 0.02, intergenic rs2121279 with 0.024 and intergenic rs2890652 with 0.09, in adult Japanese and European studies^6–8^.

Bimodality patterns were clearly visible in three CpG sites (Figs 3 and 5). Distribution of three CpG methylations were scattered, which was classified into two predefined methylation states (high-methylated and low-methylated). rs431809 is *cis-*methylation quantitative trait loci of three CpG sites in same intron. A link between genetic variation and CpG methylation represent maximum level of heritability in methylation. Differential DNA methylation changes close to BMI-associated genetic loci of *LRP1B* may drive epigenetic change in certain environments, through epigenetic regulation of chromatin structure.

Since rs431809 is located in intron 4, its functional consequences are not clear. Intronic genetic polymorphisms with certain traits might be explained by its epigenetic regulation for gene expression in target cell^23–25^. *LRP1B* is a functional tumor suppressor gene in gastric cancer, and is regulated by DNA methylation^13,16^. Epigenetic changes including DNA methylation may be important for the expression of LRP1B in certain cells, such as the human brain, thyroid gland and skeletal muscle^5^. Therefore, further analysis of obesity-related differential methylation region and genetic variations of *LRP1B*, may explain where and how sophisticated epigenetic regulations changes come from.

In conclusion, strong correlations of rs431809 genotype with closely located CpG sites trigger the epigenetic changes, which had a dynamic role in gaining weight differed by environmental factor. This study is the first study to validate the association of rs431809 in childhood obesity related to CpG methylation. Our data suggest genetic context of the *LRP1B* is directly correlated with epigenetic modification of BMI-related CpG methylation. Genetic and epigenetic interplay of *LRP1B* gene may be implicated in the prevention and therapeutic approach to childhood obesity.
